# Understanding Barriers to Cancer Pain Management: Insights From Patients and Healthcare Professionals—A Systematic Review

**DOI:** 10.1002/puh2.70247

**Published:** 2026-04-27

**Authors:** Belete Muluadam Admassie, Amanuel Sisay Endeshaw, Kumlachew Geta Belete, Diriba Teshome, Basazinew Chekol Demilew, Amare Belete Getahun, Misganew Terefe Molla, Tikuneh Yetneberk Alemayehu, Abebe Tiruneh Boled, Efrem Fenta Alemnew, Getachew Mekete Diress, Biruk Adie Admass

**Affiliations:** ^1^ Department of Anesthesia, College of Medicine and Health Sciences Bahir Dar University Bahir Dar Ethiopia; ^2^ Department of Anesthesia, College of Health Sciences Debre Markos University Debre Markos Ethiopia; ^3^ Department of Anesthesia, School of Medicine, College of Medicine and Health Sciences University of Gondar Gondar Ethiopia; ^4^ Department of Anesthesia College of Medicen and Health Science Debre Markos University Ethiopia

**Keywords:** barriers, cancer pain, healthcare providers, pain management

## Abstract

**Background:**

Cancer‐related pain is a prevalent and debilitating symptom that significantly impairs patients’ quality of life, leading to physical discomfort as well as emotional and psychological distress. Despite the availability of effective pain control strategies, cancer pain remains inadequately managed worldwide. This challenge is multifactorial, involving barriers related to patients, healthcare providers, healthcare systems, and deficiencies in coordinated multidisciplinary care.

**Methods:**

This systematic review followed the PRISMA 2020 guidelines and was registered with PROSPERO (CRD420251027582). A comprehensive search of SCOPUS, PubMed, MEDLINE, EMBASE, ScienceDirect, the Cochrane Library, and Google Scholar was conducted for studies published between 2015 and 2025. Qualitative, quantitative, and mixed‐methods studies examining barriers to cancer pain management from patient and healthcare professional perspectives were included. Study quality was assessed using the Newcastle–Ottawa Scale, and findings were synthesized narratively.

**Results:**

A total of 244 records were identified, with 23 studies meeting the inclusion criteria after screening and eligibility assessment. Overall methodological quality was good, although most studies were cross‐sectional. Four major categories of barriers were identified: patient‐related, healthcare provider‐related, healthcare system‐related, and multidisciplinary care‐related barriers. Patient‐related barriers were most frequently reported fear of opioid addiction, concerns about side effects, under‐reporting of pain, fatalistic beliefs, and cultural or religious influences. Provider‐related barriers involved inadequate training, poor pain assessment practices, opioid‐related concerns, and limited inter‐professional communication. System‐level barriers included restrictive opioid regulations, limited access to analgesics, staffing shortages, and lack of standardized protocols. Multidisciplinary barriers reflected poor care coordination, insufficient patient education, and lack of individualized pain management plans.

**Conclusion:**

Cancer pain management is hindered by interconnected barriers across patient, provider, and system levels. Addressing these challenges requires improved clinical practices, targeted professional education, balanced opioid policies, and strengthened multidisciplinary collaboration to enhance patient outcomes and quality of life.

## Graphical abstract

Cancer pain remains widely undermanaged despite available treatments, due to interconnected barriers at patient, provider, and system levels. This PRISMA‐based review (23 studies) identified key challenges including opioid fears, inadequate training, limited access to analgesics, and poor care coordination. Addressing these requires improved education, balanced policies, and stronger multidisciplinary collaboration to enhance pain control and patient quality of life.

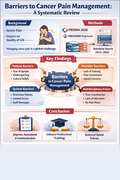

## 1 Introduction

Cancer remains a leading cause of morbidity and mortality worldwide, with an estimated 18.1 million new cases annually [1]. Globally, it is the second most common cause of death, accounting for 9.6 million deaths in 2018, the majority of which occurred in low‐income countries [2]. Even according to global cancer stastics 2020, cancer is the second leading cause of death globally, accounting for approximately 10 million deaths, with the majority occurring in low‐ and middle‐income countries. Cancer patients frequently experience severe pain, which significantly affects their quality of life and contributes to physical, emotional, and psychological distress [3]. Persistent pain, whether resulting directly from cancer or from its treatments, can disrupt recovery and remission processes, further compromising patient well‐being [4].

A meta‐analysis of 52 studies revealed that over 50% of patients worldwide experience cancer‐related pain, with certain populations, such as head and neck cancer patients, reporting even higher prevalence rates [5]. Other studies indicate that 37.9% of cancer patients experience cancer‐related pain, whereas 56.1% report nonspecific pain [6]. These figures highlight the widespread and multifaceted nature of cancer pain, underscoring the urgent need for effective management strategies.

Effective cancer pain management is often hindered by a complex interplay of factors, including patient beliefs and misconceptions, healthcare provider knowledge and practices, and systemic barriers within healthcare delivery [7]. Despite widespread acknowledgment among healthcare professionals (HCPs) of the importance of pain control, significant challenges remain. Studies indicate notable differences in perceived barriers between physicians and nurses, emphasizing the need for enhanced communication, collaboration, and standardized approaches in clinical practice [8]. Nurses, as frontline providers, play a particularly critical role in implementing both pharmacological and non‐pharmacological interventions for cancer pain management [9].

Psychological factors, such as anxiety, depression, and fatalistic beliefs, further impede effective pain management [10]. Cultural and religious influences, fear of addiction, concerns about medication tolerance, and anticipated side effects are commonly reported by patients and caregivers as barriers to optimal analgesia [11, 12]. Additionally, patient demographics, including age, gender, education level, and ethnicity, can influence pain perception and adherence to treatment [10].

HCPs also face challenges in managing cancer‐related pain due to limited training, insufficient familiarity with opioid dosing, inadequate inter‐professional collaboration, and restricted access to pain medications [13]. Previous studies have demonstrated substantial gaps in knowledge among nurses regarding opioid use, titration, and non‐pharmacological interventions, which can compromise the quality of pain management [13, 14].

Despite existing literature on cancer pain management, there remains a lack of comprehensive synthesis that simultaneously examines patient‐, provider‐, and system‐level barriers across diverse settings. Therefore, the present systematic review aims to identify and explore these multifaceted barriers, integrating perspectives from both patients and HCPs. By synthesizing current evidence, this review seeks to provide actionable insights to improve clinical practice, inform policy, and ultimately enhance patient outcomes globally.

## 2 Methods and Materials

This systematic review was conducted in accordance with the Preferred Reporting Items for Systematic Reviews and Meta‐Analyses (PRISMA) 2020 guidelines [15], and the review protocol was registered with PROSPERO (ID: 1027582) [https://www.crd.york.ac.uk/PROSPERO/view/CRD420251027582]. The methodological quality of included studies was assessed using the Newcastle–Ottawa Quality Assessment Scale (NOS) [16].

### 2.1 Search Strategy

A comprehensive search of peer‐reviewed literature published between 2015 and 2025 was conducted across multiple electronic databases, including SCOPUS, PubMed, ScienceDirect, Google Scholar, the Cochrane Library, MEDLINE, and EMBASE. The search combined keywords and Medical Subject Headings (MeSH) terms using Boolean operators (AND, OR, NOT). Search terms included: (“Cancer pain” OR “Cancer‐related pain”) AND (“Pain management” OR “Pain control”) AND (“Barriers” OR “Challenges” OR “Obstacles”). Filters for publication date (2015–2025) and language (English) were applied. The language restriction may introduce bias, which is acknowledged as a limitation of this review.

The University of Gondar library and institutional access were used to retrieve full‐text articles where necessary. This facilitated access to subscription‐based journals but did not influence the database search itself.

### 2.2 Inclusion Criteria

Studies were included if they met the following criteria: Population: Patients diagnosed with any type of cancer experiencing pain, as well as healthcare providers involved in cancer pain management. Study type: Qualitative, quantitative, or mixed‐methods studies reporting barriers to effective cancer pain management. Language: Articles published in English. Publication period: 2015–2025. Duplicate records were removed prior to screening using Endnote.

### 2.3 Screening and Study Selection

Title and abstract screening was independently conducted by two researchers (first and last authors). Full‐text screening of potentially eligible studies was performed by four researchers (second–fifth authors). Discrepancies in inclusion decisions were resolved through discussion and consensus among all researchers. Although formal inter‐rater agreement (e.g., kappa statistic) was not calculated, the consensus procedure ensured consistent selection of eligible studies.

### 2.4 Data Extraction and Synthesis

Data were extracted by four researchers, including first author, year of publication, country, study population, sample size, and identified barriers. Discrepancies during extraction were resolved by discussion between two researchers. A narrative synthesis approach was employed to summarize findings across studies, focusing on themes related to patient, healthcare provider, healthcare system, and multidisciplinary barriers. This approach was chosen because the included studies were heterogeneous in design and outcomes, making meta‐analysis inappropriate [17, 18].

### 2.5 Methodological Quality Assessment

Newcastle–Ottawa Quality Assessment Scale was used to assess the methodological quality of the studies [16]. We did a comprehensive search on literature and found that a NOS score of 7 or more can be considered a “good” study quality [19]. Therefore, we used this criterion as a cut off for good quality study. Two researchers appraise each article and two researchers reviewed each appraisal against the article full texts. When there is discrepancy during appraisal period, all researchers reviewed the full text together and reached a consensus.

## 3 Results

**FIGURE 1:**
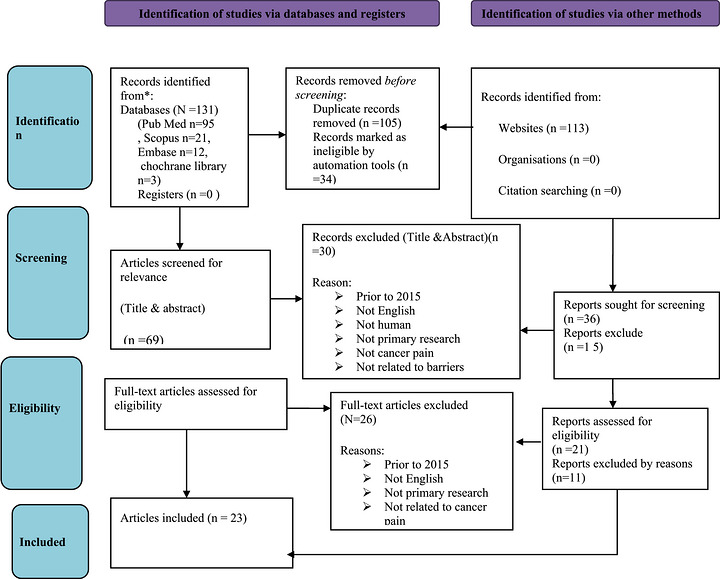
Preferred reporting items for systematic reviews and meta‐analyses (PRISMA) 2020.

The search process is illustrated in Figure 1. A total 244 articles were initially identified through searching from database and websites. After removing 139 duplicate articles, the titles and abstracts of the remaining 105 articles were screened based on the eligibility criteria. During this stage, 45 articles were excluded, remaining 60 full‐text articles for further evaluation. Out of these, 37 were excluded after a full‐text review. As a result, a total of 23 sources were deemed eligible for the review.

### 3.1 Quality Assessment

The Newcastle–Ottawa Quality Assessment Scale scoring for each study is presented in Table S1. The included articles ranged had good quality of studies. Key criteria, such as sample representativeness, nonresponse rate, ascertainment of exposure (risk factors), comparability, outcome assessment, and statistical analysis, were addressed across the studies. However, potential bias between researchers and participants was not considered. As all studies were rated good quality of study, they were deemed suitable for inclusion in the review.

### 3.2 Characteristics of Studies

Characteristics’ of studies have been provided in Table S2. First author, title, year of publication, country, study population, sample size, and identified barriers from the articles were addressed across the included studies.

### 3.3 Themes Identified Through Narrative Synthesis

Cancer pain management is still a major concern globally, impacted by a number of constraints linked to patients, HCPs, the healthcare system, and interdisciplinary methods. Research from several nations demonstrates these barriers.

### 3.4 Patient‐Related Barriers

Due to misunderstandings, anxieties, and personal beliefs, many cancer patients have difficulty managing their discomfort. Studies from Jordan [20, 21], Iraq [22], Oman [23], and Norway [24] have shown that fear of addiction and drug tolerance is still a major problem.

Cultural and religious beliefs further complicate the issue of effective cancer pain management. According to research conducted in the USA [25], patients’ religious perspectives on suffering, as well as family caregivers’ beliefs that patients should endure pain due to past actions, hinder effective pain relief. Similar results were noted in Iran [26], where patients expressed negative attitudes toward the use of analgesics and saw pain as a divine test. In Zambia [27], poor adherence to pain management was caused by use of traditional medicine and witchcraft beliefs. Additionally, research from Malaysia [28], Jordan [10], and Iran [26] revealed a fatalistic mindset in which patients accept pain as an inevitable aspect of their disease.

Marital status, gender, ethnicity, and educational level were also found to have an impact on how people perceive and manage their pain [28, 29]. On the other hand, the study also identifies important barriers to cancer pain management in South Korea [30], specifically patients’ reluctance to take opioids and their tendency to under‐report pain. In Oman [23], female patients were afraid about becoming drug‐tolerant, whereas older patients were worried about hiding their symptoms. Similar results were noted in Iraq [22]; here patients were concerned about the negative consequences of receiving pain medication (Table 1).

**TABLE 1:** Patient‐related barriers to effective cancer pain management.

Author	Year	Country	Identified barriers
Johnson et al.	2019	USA	Religious and cultural beliefs about suffering Family caregiver beliefs that patients deserve to suffer
Alaswami et al.	2024	Oman	Fear of developing tolerance to pain medications Reluctance among older patients to use analgesics Female patients’ concerns about drug tolerance
Majhool et al.	2022	Iraq	Patients’ concerns about addiction and harmful effects of pain treatment
Gunnarsdottir et al.	2017	Norway	Fear of addiction
Jho et al.	2014	South Korea	Patients’ reluctance to take opioids Under‐reporting of pain
Orujlu et al.	2021	Iran	Accepting and enduring pain as divine Negative attitudes toward analgesics Lack of knowledge about pain self‐management Neglected pain management
Kiu et al.	2021	Malaysia	Gender, ethnicity, marital status, and educational level Fatalistic mentality about pain management

### 3.5 Healthcare Providers‐Related Barriers

Effective management of cancer pain presents additional hurdles for HCPs, such as physicians, nurses, and pharmacists. Numerous studies conducted China [31, 32], Vietnam [33], Palestine [34], and Malaysia [35] have identified a lack of knowledge and training regarding pain treatment, specifically opioid therapy, as a barrier to successful pain management.

According to Jordan [36], HCPs’ concerns about opioid side effects, communication barriers, and cultural influence hinder them from prescribing and administering painkillers. A similar study in Ireland [37] found that general practitioners (GPs) had exaggerated fears of psychological addiction and lacked formal pain assessment tools.

Insufficient pain assessment and inadequate staffing were also reported as major professional‐related barriers in Saudi Arabia [38], Egypt [39], and Palestine [40]. The lack of proper inter‐professional communication further hinders pain management strategies, as seen in Jordan [41] see (Table 2).

**TABLE 2:** Healthcare providers‐related barriers for effective cancer pain management.

Author	Year	Country	Identified barriers
Saifan et al.	2019	Jordan	Fears about analgesic use and opioid side effects Communication barriers Cultural beliefs lack of knowledge
Alzghoul et al.	2022	Jordan	Misconceptions regarding analgesic side effects
Al‐Qarni et al	2024	Saudi Arabia	Inadequate pain assessment tools Concerns about opioid use Poor inter‐professional communication
Yu et al.	2022	China	Poor medication compliance Difficulty in individualized analgesia protocols Lack of multidisciplinary participation
Khalil et al.	2022	Egypt	Inadequate knowledge about pain management and opioids
McDarby et al.	2017	Ireland	Lack of knowledge about opioid analgesics Exaggerated fears of addiction Poor pain assessment tools
Liu et al.	2018	Palestine	Inadequate pain assessment lack of experience and knowledge
Kweh et al.	2022	Malaysia	Inadequate training Inconsistent knowledge about cancer pain management
Makhlouf et al.	2022	Libya	Lack of knowledge toward cancer pain management Negative attitudes
Nguyen et al.	2024	Vietnam	Knowledge deficit and negative attitudes toward opioid therapy
Samara et al.	2018	Palestine	Inadequate pain assessment
Toba et al.	2019	Palestine	Inadequate pain assessment

### 3.6 Healthcare System‐Related Barriers

Strict opioid regulations and poor access to pain medications are consistent themes in studies from multiple regions.

Regulatory restrictions on opioid distribution were identified in Egypt [29]. Furthermore, pharmacists in China [32] reported a lack of education on cancer pain management, which limited their ability to provide appropriate medications (Table 3).

**TABLE 3:** Healthcare system related barriers for effective cancer pain management.

Author	Year	Country	Identified barriers
Othman et al.	2022	Jordan	Inadequate staffing; strict opioid regulation
Ahmed et al.	2024	Egypt	Barriers related to hospital policy and medication access
Toba et al.	2019	Palestine	Strict opioid regulation
McDarby et al.	2017	Ireland	Lack of formal pain assessment tools

### 3.7 Multidisciplinary Approach‐Related Barriers

Studies indicate that an interdisciplinary approach, including better education, improved communication, and more structured policies, is necessary for effective cancer pain management. In China [31] found that poor medication compliance, difficulties in creating individualized pain protocols, and insufficient multidisciplinary participation contributed to inadequate pain control. Similar findings were observed in Malaysia [28], where a lack of patient education and communication between medical staff and patients led to suboptimal pain management (Table 4).

**TABLE 4:** Multidisciplinary approach‐related barriers to effective cancer pain management.

Author	Year	Country	Identified barriers
Saifan et al.	2019	Jordan	Communication barriers between healthcare teams
Jho et al.	2024	South Korea	Under‐reporting of pain by patients and lack of coordination in pain management
Othman et al.	2022	Jordan	Lack of communication between patients and medical staff
Yu et al.	2022	China	Insufficient multidisciplinary collaboration in pain management
Kiu et al.	2021	Malaysia	A lack of patient education and communication between medical staff and patients

## 4 Discussion

This systematic review highlights the multifaceted barriers to effective cancer pain management, encompassing patient‐related, healthcare provider‐related, healthcare system‐related, and multidisciplinary‐related factors. Across diverse global contexts, several consistent themes emerged, yet variations between regions indicate the influence of cultural, social, and regulatory environments.

### 4.1 Patient‐Related Barriers

Fear of opioid addiction, concerns about drug tolerance, and reluctance to report pain remain dominant patient‐related barriers, consistent with studies from Jordan, Iraq, Oman, Norway, and South Korea [20, 21, 22, 23, 24, 30]. Cultural and religious beliefs further influence pain management behaviors, with fatalistic attitudes and acceptance of pain as inevitable observed in Malaysia, Jordan, Iran, and the USA [10, 25, 26, 28]. These findings underscore the importance of culturally sensitive education and counseling interventions to improve adherence to pain management protocols. Although many studies reported similar patient‐related barriers, regional differencessuch as reliance on traditional medicine in Zambiahighlight the need for context‐specific strategies.

### 4.2 Healthcare Provider‐Related Barriers

HCPs face challenges due to limited knowledge and training on cancer pain management, particularly regarding opioid use, dosing, and non‐pharmacological interventions [31, 32, 33, 34, 35]. Concerns about side effects, communication barriers, and cultural influences were also reported [36, 37]. Staffing shortages and inadequate interdisciplinary collaboration further compound these challenges. Notably, some studies demonstrated that even when providers were aware of guidelines, implementation gaps persisted due to resource constraints and organizational factors, emphasizing the need for ongoing professional development and system support.

### 4.3 Healthcare System‐Related Barriers

System‐level barriers, including strict opioid regulations, restricted access to pain medications, and lack of standardized protocols, were consistently reported across regions [29, 32, 40]. Regulatory constraints may limit clinicians’ ability to provide optimal analgesia, whereas variations in policy and resource allocation between countries highlight the importance of aligning clinical guidelines with national and institutional regulations.

### 4.4 Multidisciplinary Approach‐Related Barriers

The review emphasizes the need for multidisciplinary approaches to address cancer pain effectively. Poor communication between professionals, inadequate patient education, and limited integration of individualized pain protocols were recurrent barriers [28, 31]. Strengthening interdisciplinary collaboration, for example, through joint care planning and shared decision‐making, can enhance adherence to pain management regimens. Clear role definitions among nurses, physicians, and pharmacists, combined with structured educational programs, may mitigate both patient‐ and provider‐related barriers.

### 4.5 Analytical Synthesis and Implications

Although the literature consistently identifies opioid fear, fatalism, and cultural beliefs as barriers, the magnitude and context differ across regions. Integrating these findings, it is evident that interventions must be both evidence‐based and culturally tailored. Clinically, nurses play a central role in assessment, education, and patient advocacy; equipping them with ongoing training can directly improve pain control. Policy implications include revisiting restrictive opioid regulations and promoting institutional guidelines to facilitate timely and adequate analgesia. Educational initiatives targeting both healthcare providers and patients are essential to overcome misconceptions and improve communication.

### 4.6 Limitations and Critical Reflection

This review has several limitations. Most included studies were cross‐sectional or qualitative, limiting causal inference. Regional disparities in healthcare infrastructure and cultural norms may influence findings, reducing generalizability. Some studies did not adequately address bias or confounding factors, and reporting of sample characteristics varied. Additionally, the review itself is limited by language and publication restrictions, potentially excluding relevant evidence. Future research should focus on longitudinal studies and interventions that test multidisciplinary strategies in diverse settings.

## 5 Conclusion and Recommendation

This systematic review underscores the multifaceted barriers to effective cancer pain management, encompassing patient‐related factors, HCPs challenges, healthcare system limitations, and the critical need for multidisciplinary collaboration. Fear of addiction, cultural and religious beliefs, inadequate knowledge among healthcare providers, regulatory restrictions on opioid use, and poor interdisciplinary coordination were consistently identified as major obstacles. To address these barriers, we recommend the following actionable strategies:

**Clinical practice**: Implement standardized pain assessment protocols, promote interdisciplinary case discussions, and ensure timely access to appropriate analgesics, including opioids where indicated.

**Education and training**: Develop ongoing, evidence‐based training programs for healthcare professionals focused on pain assessment, opioid use, non‐pharmacological interventions, and culturally sensitive communication with patients and caregivers.

**Policy and system‐level interventions**: Streamline opioid regulations to balance safety and access, improve resource allocation to ensure adequate staffing, and establish policies that encourage multidisciplinary collaboration and patient‐centered care.

By integrating these strategies, healthcare systems can improve patient outcomes, enhance adherence to pain management protocols, and ensure that individuals with cancer receive comprehensive and effective pain relief.

## Author Contributions

**Belete Muluadam Admassie** authors share primary authorship of this manuscript, They were both actively involved in formulating the research question, developing the methodology, and conducting data collection and analysis. They also co‐led the manuscript writing process. **Biruk Adie Admass, Amanuel Sisay Endeshaw**, **Kumlachew Geta Belete**, and **Diriba Teshome** authors supported data extraction and analysis and helped draft the manuscript. The **Basazinew Chekol Demilew** and **Amare Belete Getahun** authors contributed to the methodology, data analysis, and interpretation of the results. The **Misganew Terefe Molla**, **Tikuneh Yetneberk Alemayehu**, **Abebe Tiruneh Boled**, and **Efrem Fenta Alemnew** authors provided critical reviews of the manuscript and assisted in writing the implications section. All authors satisfy the authorship criteria, having (i) significantly contributed to the work, (ii) participated in drafting and/or critically revising the manuscript, (iii) approved the final version for publication, and (iv) been sufficiently involved to take public responsibility for its content.

## Funding

The authors have nothing to report.

## Ethics Statement

The authors have nothing to report.

## Conflicts of Interest

The authors declare no conflicts of interest.

## Supporting information




**Supplementary File1**: puh270247‐sup‐0001‐TableS1.docx

**Supplementary File2**: puh270247‐sup‐0002‐TableS2.docx

## Data Availability

The data that support the findings of this study are available on request from the corresponding author. The data are not publicly available due to privacy or ethical restrictions.
