# Supplementary material for: Understanding Barriers to Cancer Pain Management: Insights From Patients and Healthcare Professionals—A Systematic Review
Source: Public Health Chall. 2026 Apr 27;5(2):e70247. doi: 10.1002/puh2.70247 (PMC13114765; doi:10.1002/puh2.70247)
Supplement: Supplementary file 1 — Supplementary File1: puh270247‐sup‐0001‐TableS1.docx [file PUH2-5-e70247-s002.docx]

**Supplemental Table 1: Newcastle - Ottawa Quality Assessment Scale scoring**

| **Sources** | **Representativeness of the sample**  **(Max 3⃰)** | **Non-respondents**  **Max (1⃰)** | **Ascertainment of the exposure (risk factor)(1⃰)** | **Comparability**  **(max 2⃰)** | **Assessment of the outcome**  **(Max 2⃰)** | **statistical test**  **Max (1⃰)** | **Score** |
| --- | --- | --- | --- | --- | --- | --- | --- |
| Johnson et al. (2019) | ** | Not tell | Not tell | ** | ** | * | 7/10 |
| Al-Ghabeesh et al., 2019 | * | Not tell | * | ** | ** | * | 7/10 |
| Saifan et al. (2019) | *** | * | * | * | * | * | 8/10 |
| Alaswami et al., 2024 | ** | Not tell | Not tell | ** | ** | * | 7/10 |
| Majhool et al., 2022 | ** | Not tell | * | ** | ** | * | 8/10 |
| Alzghoul et al., 2022 | *** | * | * | * | * | * | 8/10 |
| Gunnarsdottir et al., 2017 | *** | Not tell | * | * | * | * | 7/10 |
| Yu et al., 2022 | *** | * | * | ** | ** | * | 9/10 |
| Othman et al., 2022 | *** | Not tell | * | * | ** | * | 8/10 |
| Khalil et al., 2022 | ** | Not tell | Not tell | ** | ** | * | 7/10 |
| Ahmed et al., 2024 | ** | Not tell | * | * | ** | * | 7/10 |
| Mulonda et al., 2023 | * | Not tell | * | ** | ** | * | 7/10 |
| McDarby et al., 2017 | ** | * | * | * | * | * | 7/10 |
| Orujlu et al., 2021 | * | Not tell | * | ** | ** | * | 7/10 |
| Kiu et al., 2021 | ** | Not tell | * | ** | * | * | 7/10 |
| Liu et al., 2025 | ** | * | * | * | * | * | 7/10 |
| Samara et al., 2018 | ** | * | * | * | ** | * | 8/10 |
| Kweh et al., 2022 | *** | Not tell | * | * | * | * | 7/10 |
| Toba et al., 2019 | ** | * | * | ** | ** | * | 9/10 |
| Makhlouf et.al,2022 | ** | * | * | ** | * | * | 8/10 |
| Nguyen et al., 2024 | ** | Not tell | Not tell | ** | ** | * | 7/10 |
| Lee Kiu, et.al, 2021 | ** | Not tell | * | ** | ** | * | 8/10 |
| **Hyun Jung Jho et al. (2014)** | *** | Not tell | Not tell | ** | ** | * | 8/10 |
