# Supplementary material for: Understanding Barriers to Cancer Pain Management: Insights From Patients and Healthcare Professionals—A Systematic Review
Source: Public Health Chall. 2026 Apr 27;5(2):e70247. doi: 10.1002/puh2.70247 (PMC13114765; doi:10.1002/puh2.70247)
Supplement: Supplementary file 2 — Supplementary File2: puh270247‐sup‐0002‐TableS2.docx [file PUH2-5-e70247-s001.docx]

**Supplemental Table 2: Summary of Studies Reporting Barriers to Effective Cancer Pain Management**

| Author (Year, Country) | Study Title (Shortened) | Design | Sample | Key Barriers Identified |
| --- | --- | --- | --- | --- |
| Johnson et al. (2019, USA) | Perceptions of HCPs on CPM | Comparative | 26 HCPs | Patients’ cultural/religious beliefs; family attitudes about suffering |
| Al-Ghabeesh et al. (2019, Jordan) | Barriers from Patients and Families | Qualitative | 10 patients, 10 caregivers | Limited knowledge; reliance on cultural/religious coping methods |
| Saifan et al. (2019, Jordan) | HCP Factors in Pain Management | Descriptive correlational | 473 HCPs | Opioid side-effect concerns; communication issues; limited training |
| Alaswami et al. (2024, Oman) | Barriers in Omani Cancer Patients | Cross-sectional | 68 patients | Fear of drug tolerance; hesitance among older and female patients |
| Majhool et al. (2022, Iraq) | Patient Attitudes on Pain Relief | Cross-sectional | 130 patients | Concerns over addiction and treatment side effects |
| Alzghoul et al. (2022, Jordan) | Patient Barriers per Nurses | Cross-sectional | 307 nurses | Misconceptions about analgesic risks |
| Gunnarsdottir et al. (2017, Norway) | Attitudinal Obstacles in CPM | Cross-sectional | 555 patients | Fear of opioid dependence |
| Al-Qarni et al. (2024, KSA) | Literature Review on CPM Issues | Review | N/A | Tool limitations, opioid concerns, poor interprofessional collaboration |
| Jho et al. (2014, Korea) | CPM Barriers in Korean HCPs | Cross-sectional | 149 physicians, 284 nurses | Patient reluctance, under-reporting of pain |
| Yu et al. (2022, China) | CPM Practices in Oncology Units | Cross-sectional | 1262 HCPs | Noncompliance, complex pain protocols, lack of teamwork |
| Othman et al. (2022, Jordan) | Oncology Nurses on CPM | Cross-sectional | 502 nurses | Inadequate staffing, opioid regulation, poor communication |
| Khalil et al. (2022, Egypt) | Nurses' Pain Management Practices | Descriptive correlational | 122 nurses | Gaps in knowledge about opioids and pain management |
| Ahmed et al. (2024, Egypt) | Patient Views in Critical Care | Descriptive | 150 patients | Drug fears, hospital rules, poor self-management |
| Mulonda et al. (2023, Zambia) | HCPs on Cultural Barriers | Qualitative | 10 doctors | Traditional beliefs, spiritual interpretations, knowledge gaps |
| McDarby et al. (2017, Ireland) | Irish GPs on CPM Challenges | Cross-sectional | 138 GPs | Knowledge gaps on opioid dosing, fear of addiction, poor pain assessment |
| Orujlu et al. (2021, Iran) | Patients' Beliefs About Pain | Qualitative | 14 patients | Spiritual acceptance, distrust of medications, lack of knowledge |
| Kiu et al. (2021, Malaysia) | Demographic Influences on Barriers | Cross-sectional | 133 patients | Gender, education, marital status, ethnicity impact attitudes |
| Liu et al. (2025, China) | Pharmacists’ Perspective on CPM | Cross-sectional | 339 pharmacists | Limited formal education in pain control |
| Samara et al. (2018, Palestine) | Physicians’ CPM Practices | Cross-sectional | 109 physicians | Experience gaps, weak assessment, limited training |
| Kweh et al. (2022, Malaysia) | Doctors’ Knowledge on CPM | Cross-sectional | 321 doctors | Training deficits, mixed perceptions of opioid use |
| Toba et al. (2019, Palestine) | Nurses' CPM Perceptions | Cross-sectional | 220 nurses | Pain underassessment, opioid access issues |
| Makhlouf et al. (n.d., Libya) | HCP Attitudes Toward CPM | Cross-sectional | 152 HCPs | Poor knowledge and negative perceptions |
| Nguyen et al. (2024, Vietnam) | Opioid Therapy Barriers | Cross-sectional | 480 HCPs | Misinformation and biases about opioids |
| Doreen Kiu et al. (2021, Malaysia) | Psychosocial Patient Barriers | Cross-sectional | 133 patients | Fatalistic views, limited education, communication challenges |
